# What's new in the pathogeneses and triggering factors of bullous pemphigoid

**DOI:** 10.1111/1346-8138.16654

**Published:** 2022-11-22

**Authors:** Hideyuki Ujiie

**Affiliations:** ^1^ Department of Dermatology, Faculty of Medicine and Graduate School of Medicine Hokkaido University Sapporo Japan

**Keywords:** BP180, BP230, epitope spreading, immune checkpoint inhibitor, regulatory T cell

## Abstract

Bullous pemphigoid (BP) is a subepidermal blistering disease induced by autoantibodies to type XVII collagen (COL17, also called BP180) and BP230. Previous studies using patients' samples and animal disease models elucidated the complement‐dependent and complement‐independent pathways of blister formation, the pathogenic roles of immune cells (T and B cells, macrophages, mast cells, neutrophils, eosinophils), and the pathogenicity of IgE autoantibodies in BP. This review introduces the recent progress on the mechanism behind the epitope‐spreading phenomenon in BP, which is considered to be important to understand the chronic and intractable disease course of BP, and the pathogenicity of anti‐BP230 autoantibodies, mainly focusing on studies that used active disease models. To clarify the pathogenesis of BP, the mechanism behind the breakdown of immune tolerance to BP antigens should be investigated. Recent studies using various experimental models have revealed important roles for regulatory T cells in the maintenance of self‐tolerance to COL17 and BP230 as well as in the suppression of inflammation triggered by the binding of antibodies to COL17. Notably, physical stresses such as trauma, thermal burns, bone fractures, irradiation and ultraviolet exposure, some pathologic conditions such as neurological diseases and hematological malignancies, and the use of dipeptidyl peptidase‐IV inhibitors and immune checkpoint inhibitors have been reported as triggering factors for BP. These factors and certain underlying conditions such as genetic background, regulatory T‐cell dysfunction or aging might synergistically affect some individuals and eventually induce BP. Further studies on the breakdown of self‐tolerance and on the identification of key molecules that are relevant to blister formation and inflammation may expand our understanding of BP's etiology and may lead to the development of novel therapeutic approaches.

## 1 INTRODUCTION

Bullous pemphigoid (BP) is the most common autoimmune blistering disease. It characteristically affects the elderly, and recent studies have reported a trend of increased incidence of BP.^1^ Clinically, it is characterized by tense blisters, urticarial plaques, and erythema on the whole body; histologically, subepidermal blisters with prominent eosinophilic infiltration are usually observed.^2^ Autoantibodies in BP react with two structural components of the dermal‐epidermal junction (DEJ): type XVII collagen (COL17, also called BP180 or BPAG2) and BP230 (also called dystonin or BPAG1).^3^ COL17 is a hemidesmosomal transmembrane protein that spans the lamina lucida and projects into the lamina densa of the DEJ.^4^, ^5^, ^6^ The extracellular portion of COL17 contains 15 collagenous domains that are separated from one another by noncollagenous domains.^5^ The noncollagenous 16A (NC16A) domain is located at the membrane‐proximal extracellular region of COL17 and is preferentially recognized by autoantibodies in BP.^7^, ^8^ In fact, 80%–90% of BP sera react with the NC16A domain of COL17.^7^, ^9^, ^10^ Previous studies have demonstrated that the serum levels of autoantibodies to the NC16A domain of COL17 correlate with BP disease activity.^7^, ^8^, ^11^, ^12^ The passive transfer of IgG antibodies to the NC16A domain of human COL17 or its murine counterpart into neonatal mice has been shown to induce subepidermal separation.^13^, ^14^ Thus, the NC16A domain of COL17 contains the major pathogenic epitope for BP, and an ELISA using recombinant NC16A protein is widely used for detecting and quantifying BP autoantibodies. Notably, the anti‐COL17 antibody can also recognize other epitopes on COL17, other than the NC16A domain. To detect all antibodies to COL17, we established an ELISA that uses mammalian cell‐derived recombinant human full‐length COL17 protein, which improved the BP autoantibody detection rate from 82.6% (NC16A ELISA alone) to 94.2% (combined use of NC16A ELISA and full‐length COL17 ELISA).^15^ Intriguingly, non‐NC16A BP in which the antibodies were detected by the full‐length COL17 ELISA alone significantly showed a noninflammatory phenotype that is characterized by few erythema and few eosinophilic infiltration in peri‐blistering dermis and likely to receive dipeptidyl peptidase‐IV (DPP‐4) inhibitors (DPP‐4i) before BP onset.^15^

BP230, a cytoplasmic component of hemidesmosomes that belongs to the plakin family, is another autoantigen of BP, and it is targeted by autoantibodies in about 50%–80% of BP cases.^16^, ^17^, ^18^ The autoantibodies preferentially target the C‐terminal domain of BP230.^16^, ^17^ Although several studies have noted the pathogenicity of autoantibodies to BP230,^19^, ^20^ it remains uncertain whether anti‐BP230 autoantibodies directly contribute to blister formation or whether they are just by‐products of epitope spreading associated with disease extension.

Recent clinical research and ex vivo and in vivo experiments have gradually elucidated the pathomechanisms and triggering factors of BP. The present review focuses on the epitope‐spreading phenomenon in BP, the pathogenicity of autoantibodies to BP230, the impact of regulatory T‐cell (Treg) dysfunction on BP, and the triggering factors for BP.

## 2 EPITOPE SPREADING IN BP

Epitope spreading is a phenomenon in which the targets of cellular and/or humoral immune responses can extend from the initial dominant epitope to other epitopes on the same protein (intramolecular epitope spreading) or to other proteins in the same tissue (intermolecular epitope spreading) over time.^21^, ^22^ Some autoimmune disorders such as multiple sclerosis^23^ and myasthenia gravis^24^ are known to show intramolecular epitope spreading. It is well known that epitope spreading frequently occurs in BP. In vivo experiments using a human COL17‐expressing skin‐grafted BP mouse model showed that IgG antibodies to human COL17 initially react to the extracellular domains and subsequently target additional extracellular domains and intracellular domains.^25^ A prospective multicenter study demonstrated that 17 of 35 (49%) BP patients showed epitope spreading that preferentially occurred at an early stage of the disease and was associated with disease severity.^26^ Of note, three of those 17 cases showed intermolecular epitope spreading from COL17 to BP230, but the reactivity to BP230 never preceded that to COL17.^26^ Thus, epitope spreading has been demonstrated in both experimental murine BP and human BP.

**FIGURE 1:**
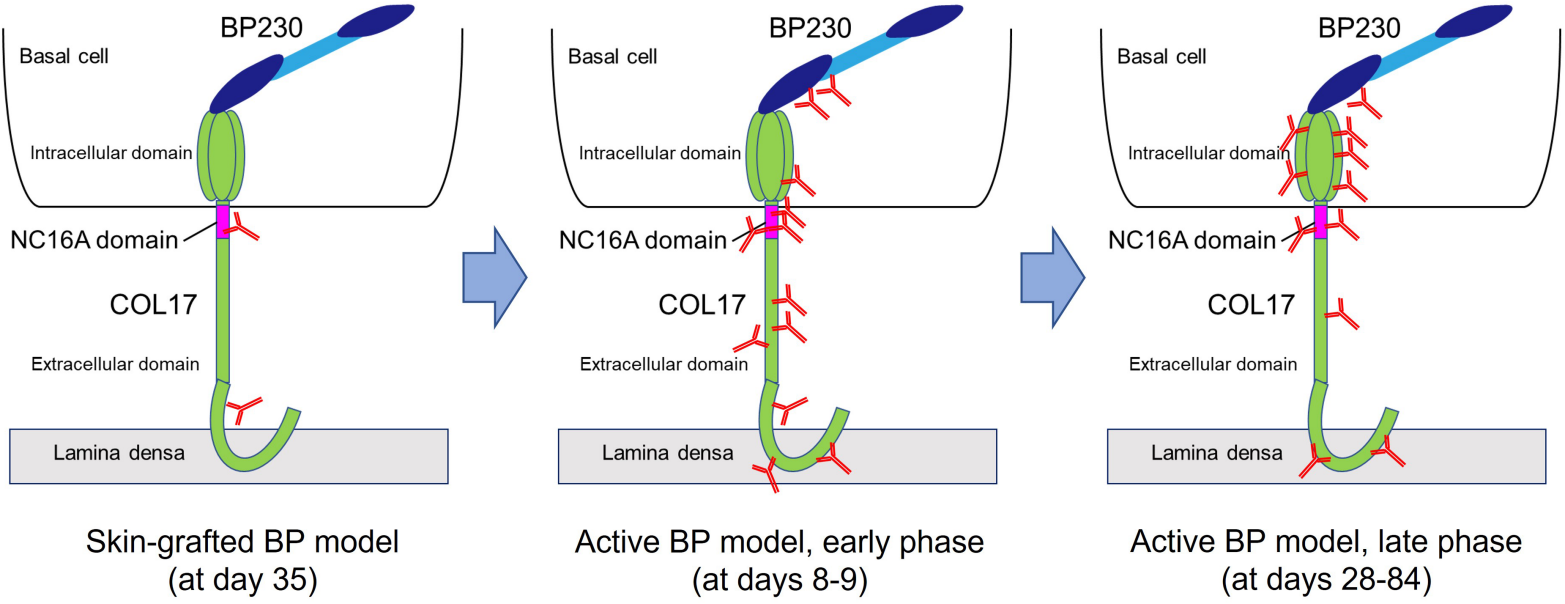
Epitope‐spreading in the skin‐grafted mouse model and in the active bullous pemphigoid (BP) mouse model. A schematic of COL17, BP230, and antibodies. In the skin‐grafted mouse model at day 35, the antibodies mainly react to the NC16A domain and weakly react to the extracellular domain epitopes of COL17. After the adoptive transfer of immunized spleen cells, there is intramolecular epitope‐spreading to extracellular domain epitopes and to the outer portion of the intracellular domain of COL17, and there is intermolecular epitope‐spreading to BP230. This is followed by intramolecular epitope‐spreading to the inner portion of the intracellular domain of COL17.^28^

However, questions remain, for example whether the T‐ and B‐cell interactions for different epitopes of COL17 occur at different time points and whether an immune response to the NC16A domain of COL17 actually triggers intramolecular epitope spreading to other epitopes of COL17 and/or intermolecular epitope spreading to other hemidesmosomal antigens. To address these issues, we used an active BP mouse model that we had established.^27^ First we immunized wild‐type mice by grafting them with human COL17‐expressing transgenic mouse skin (the skin‐grafted BP model). Then, the immunized spleen cells were adoptively transferred into immunodeficient *Rag‐2* ^*−/−*^/COL17‐humanized (*COL17* ^*m−/−,h+*^) mice (the active BP model). This model continuously produces IgG antibodies to human COL17 in a CD4^+^ T‐cell‐dependent and B‐cell‐dependent manner and reproduces the BP disease phenotype. Alternatively, NC16A peptide‐immunized spleen cells were also transferred into the recipient mice in some experiments. By using these models, we demonstrated that the production of antibodies to the extracellular domains of COL17 precedes that to the intracellular domains, especially to the inner portion of the intracellular domain.^28^ Both wild‐type mice immunized with NC16A peptides and the recipients of those spleen cells produced IgG antibodies to intracellular domains and extracellular domains, showing intramolecular epitope spreading from the NC16A domain to other epitopes of COL17. Furthermore, the active BP model mice show intermolecular epitope spreading from human COL17 to murine BP230. These results suggest that the immune response to the extracellular domains of COL17, especially to the NC16A domain, triggers intramolecular and intermolecular epitope spreading to intracellular domains of COL17 and to murine BP230 (Figure 1). In addition, the blockade of CD40–CD40 ligand interaction soon after the adoptive transfer of spleen cells was found to suppress the production of antibodies to the NC16A domain but not to intracellular domains in the recipients, suggesting the sequential activation from T and B cells against the extracellular domains, including the NC16A domain, to those against intracellular domains in vivo.

Conversely, we recently treated a unique case of BP that showed intermolecular epitope spreading from BP230 to COL17.^29^ The patient initially had a localized bullous lesion on the right thigh, and anti‐BP230 antibodies but not anti‐full‐length COL17 antibodies nor anti‐COL17 NC16A antibodies were detected at that time. A few months later, the patient developed multiple blisters on the whole body, predominantly on the hands and soles, that resembled dyshidrosiform pemphigoid, and anti‐COL17 NC16A antibodies became detectable in addition to anti‐BP230 antibodies. Thus, this case suggests that intermolecular epitope spreading from BP230 to the NC16A domain of COL17 may occur in BP, although there is the possibility that antibodies to COL17 that were undetectable by our current experimental system may exist at onset. Further studies are necessary to elucidate the mechanism behind this unique phenomenon.

## 3 THE PATHOGENICITY OF AUTOANTIBODIES TO BP230

Although the autoantibody to BP230 is frequently detected in BP as described above, its pathogenicity is controversial because BP230 is an intracellular molecule. A previous study demonstrated that the subcutaneous injection of anti‐BP230 antibodies isolated from a rabbit that was immunized with small peptides of the C‐terminus domain of human BP230 induced blister formation in the neonatal mice, suggesting the in vivo pathogenicity of anti‐BP230 antibodies.^20^ Haeberle et al. demonstrated the pathogenicity of the anti‐BP230 antibody using scurfy mice that lack functional Treg cells due to *Foxp3* gene mutation and that develop autoimmunity in multiple organs, including the skin.^30^ They generated hybridomas using lymphocytes from scurfy mice and obtained a monoclonal antibody to BP230. The passive transfer of the monoclonal antibody induced subepidermal blisters in neonatal mice.

Recently, Makita et al.^31^ generated a novel active mouse model that produces the anti‐BP230 antibody in vivo. First, they generated BP230 conditional knockout mice whose BP230 knockout is restricted to keratin 5‐expressing epithelial cells. Then, those mice were immunized with the C‐terminal portion of BP230, and the spleen cells were adoptively transferred into *Rag2* ^*−/−*^ mice. The recipient mice developed subepidermal blisters on the feet and tails, with the linear deposition of IgG at the DEJ. Interestingly, when surface wounds were made on the dorsum of the mice after the adoptive transfer, the wounded recipient mice developed earlier and more severe BP‐like symptoms. These findings suggest that the anti‐BP230 antibody induces subepidermal blisters in vivo and that trauma provokes blister occurrence.

From the clinical aspect, Hayakawa et al.^32^ reported the clinical and immunological features of patients with anti‐BP230 but not anti‐COL17 antibodies (BP230‐BP). They collected 14 cases of BP230‐BP and compared them with COL17‐BP230‐BP (*n* = 17) and COL17‐BP (*n* = 14). The BP230‐BP cases showed a smaller disease area, a lower score on the Bullous Pemphigoid Disease Area Index (BPDAI), and a lower peripheral eosinophil count compared to other groups. Thus, BP230‐BP tends to present a mild clinical course, with less inflammation. We recently analyzed IgG subclasses and complement deposition in six cases of BP230‐BP and found that IgG4, without complement activation ability, was clearly deposited at the DEJ of the skin in all cases but that the deposition of IgG1 and IgG3, with high complement activation ability, was faint or negative in all of the BP230‐BP cases.^33^ According to these findings, the deposition of complement C3 tended to be weaker in BP230‐type BP than in BP180‐BP230‐type BP. From these results, we concluded that the mild clinical phenotype of BP230‐BP may correlate with the weaker deposition of IgG1, IgG3, and complement in the skin.^33^ We also reported a case of mucous membrane pemphigoid^34^ and a case of nonbullous pemphigoid^35^ who had anti‐BP230 but not anti‐COL17 antibodies.

**FIGURE 2:**
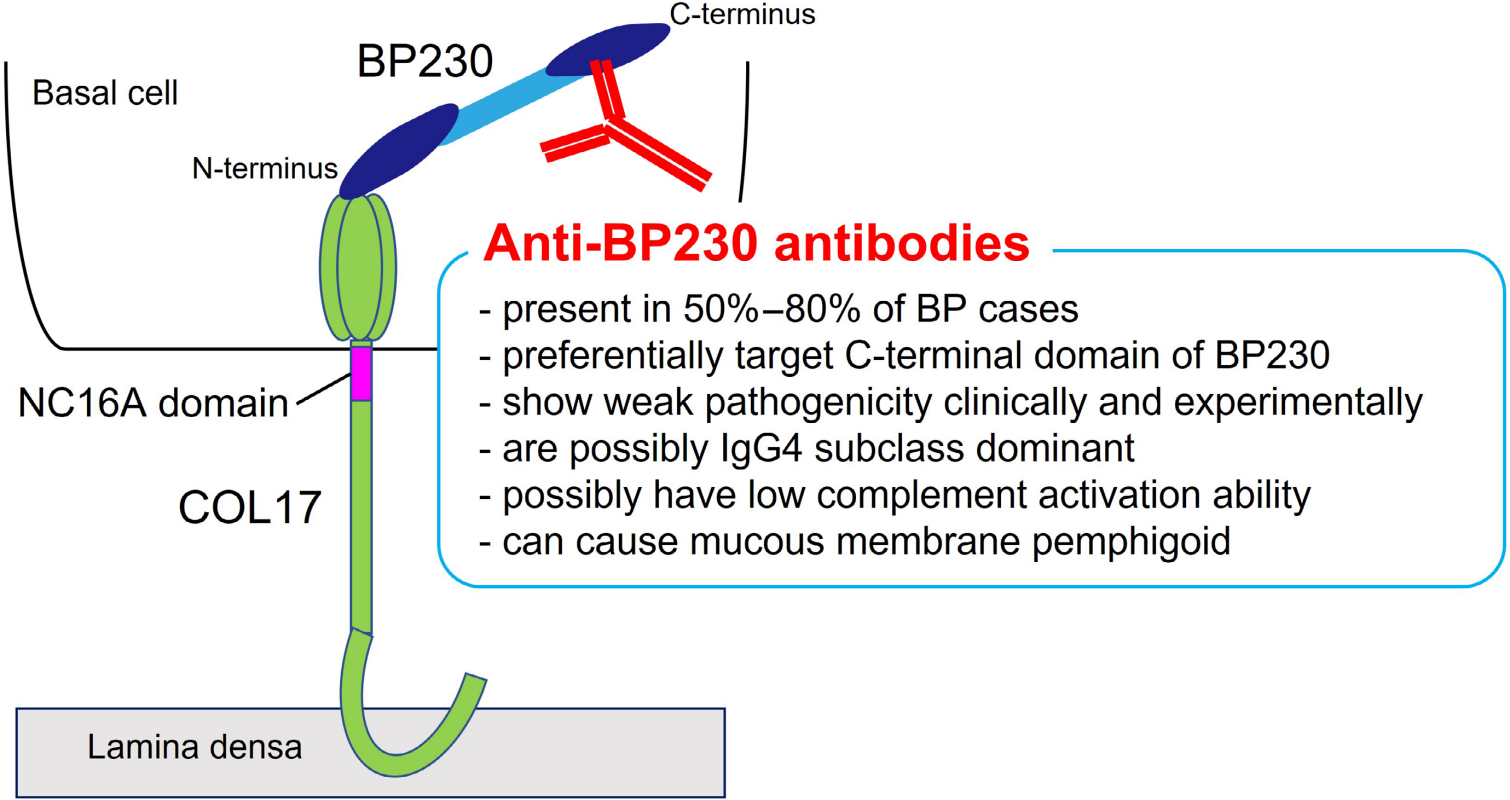
Characteristics of the anti‐BP230 antibody. A schematic of COL17, BP230, and the anti‐BP230 antibody. BP, bullous pemphigoid.

These experimental and clinical findings strongly suggest a certain role for anti‐BP230 antibodies in BP development, but the pathogenicity may be lower than that of anti‐COL17 antibodies (Figure 2).

## 4 THE IMPACT OF REGULATORY T‐CELL DYSFUNCTION IN BP

What is the mechanism behind the breakdown of self‐tolerance in BP? Self‐tolerance is maintained by central tolerance and peripheral tolerance, and *Foxp3* ^*+*^ Treg cells are the main player in peripheral tolerance. Treg cells keep autoreactive T cells that escape clonal deletion in the thymus from activating and expanding at the periphery. Treg cells use multiple suppressor mechanisms. They secrete the immuno‐inhibitory molecules IL‐10, IL‐35, TGF‐β, and cytotoxic proteins such as granzyme and perforin. Treg cells also actively engage in removing vital components from cells of other types, including antigens (peptide–MHC Class II) via T‐cell receptors, costimulatory molecules CD80 and CD86 via CTLA‐4, IL‐2 via CD25, and inflammatory signaling molecules such as ATP via CD39 and CD73 on their surface.^36^ Mutations in *Foxp3* result in fatal immune dysregulation, polyendocrinopathy, enteropathy, X‐linked (IPEX) syndrome in humans,^37^ and a lymphoproliferative syndrome in mice (scurfy mice).^38^, ^39^

Treg cells have been reported to play important roles in the skin, such as the mitigation of skin inflammation on repeated antigen exposure by skin‐resident memory Treg cells,^40^ the acceleration of wound healing,^41^ the promotion of immune tolerance to skin commensal microbes,^42^ the orchestration of hair follicle regeneration,^43^ and the promotion of hair follicle stem cell differentiation during skin barrier repair.^44^ Thus, the function of Treg cells in maintaining skin homoeostasis has been gradually elucidated. Clinically, there are many reports regarding the relevance of Treg cells to various autoimmune skin disorders such as alopecia areata, vitiligo, pemphigus, pemphigoid, and systemic sclerosis.^45^

**FIGURE 3:**
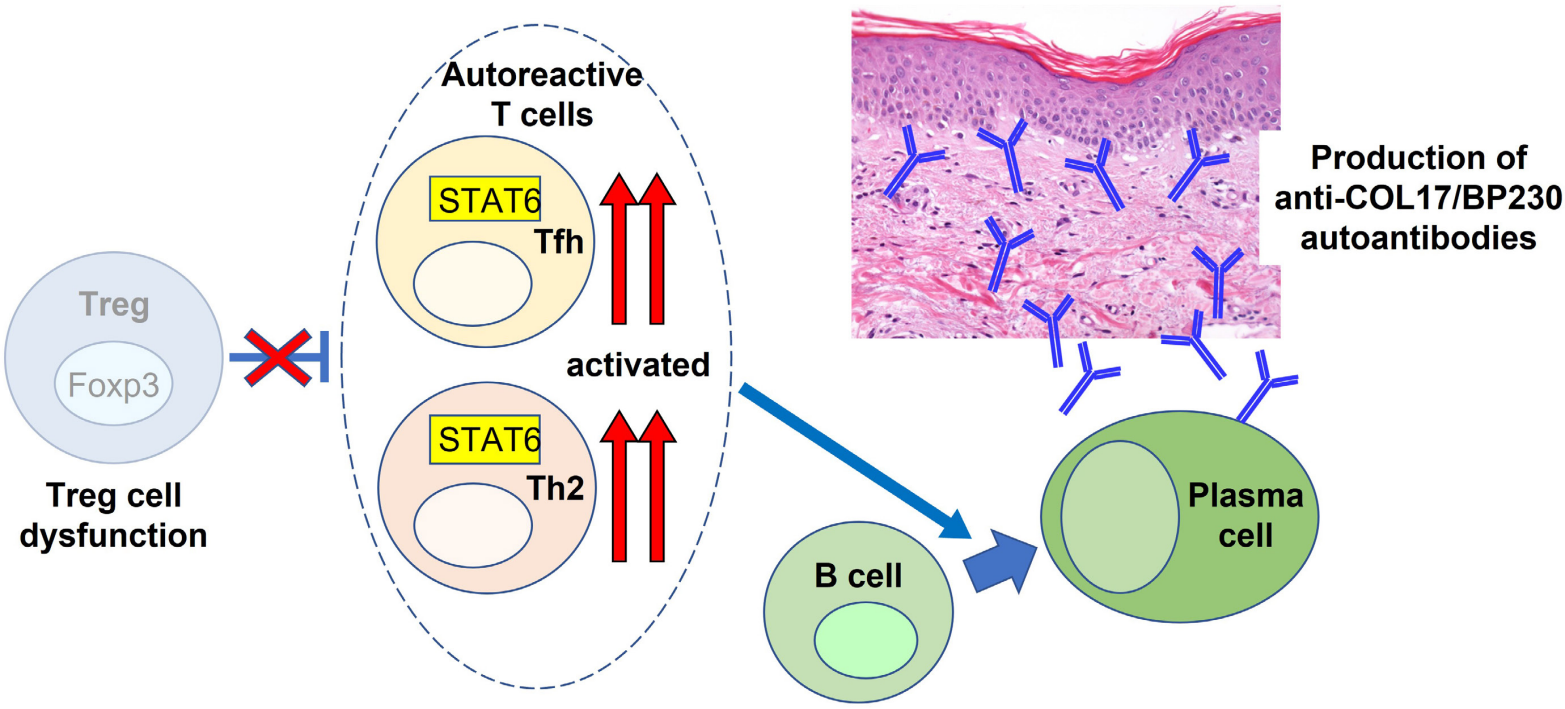
Treg cell dysfunction (*Foxp3* mutation) induces autoantibodies to COL17 and BP230. This dysfunction triggers the activation of autoreactive CD4^+^ Th2 and follicular helper T (Tfh) cells mediated by STAT6, which helps B cells to differentiate into autoantibody‐secreting plasma cells. Some of these plasma cells can produce IgM and IgG autoantibodies to COL17 and BP230.

To examine the pathogenic role of Treg cells in BP, we investigated scurfy mice, which lack functional Treg cells due to a mutation in *Foxp3*.^46^ Direct and indirect immunofluorescence studies showed that scurfy mice develop IgG autoantibodies to the DEJ of skin that has been class‐switched from IgM within 12 days after birth. Immunoblotting using recombinant proteins of COL17 and BP230 detected autoantibodies to those proteins in scurfy sera. However, autoantibodies in scurfy sera have no reactivity to the NC14A domain of murine COL17, the domain that is responsible for subepidermal blister formation. Subcutaneous injection of polyclonal IgG autoantibodies from scurfy sera did not induce skin fragility in neonatal mice. Furthermore, a CD4^+^ T‐cell‐transfer model revealed that CD4^+^ T cells from scurfy mice induced the production of autoantibodies to COL17 and BP230 in recipient *Tcrbd* ^−*/*−^ mice that lack T cells but have B cells. *Stat6* ^−*/*−^ scurfy mice, whose central signaling pathway for Th2 is knocked out, showed the reduced production of these autoantibodies and reduced numbers of follicular helper T cells, which resulted in the mitigation of skin changes. We also identified autoantibodies to COL17 in patients with IPEX syndrome, the human counterpart of scurfy mice. In conclusion, Treg cell dysfunction spontaneously induces autoantibodies to BP antigens in mice and humans (Figure 3). Haeberle et al.^30^ also demonstrated that scurfy mice spontaneously generate pathogenic anti‐BP230 autoantibodies and develop subepidermal blisters in vivo. These findings strongly suggest that Treg cells play an important role in maintaining self‐tolerance to BP antigens in steady state. Bieber et al. examined Treg cell function in BP by using an anti‐mouse COL17 IgG passive‐transfer model.^47^ They injected anti‐mouse COL17 IgG into Foxp3^DTR‐eGFP^ (DEREG) mice and depleted Treg cells by using a diphtheria toxin. They found that the depletion of Treg cells induced severe disease progression associated with an increase in leukocyte dermal infiltration, while IgG and C3 deposition at the DEJ were unaffected. The depletion of Treg cells also induced higher gene expression of IL‐10, IFN‐g, IL‐4, IL‐13, and CXCL‐9 in lesional skin. These results suggest that Treg cells control myeloid cell‐mediated skin inflammation in an experimental BP model.

But what about Treg cells in BP patients? Rensing‐Ehl et al. reported that the number and the suppressive function of circulating CD4^+^CD25^+^Foxp3^+^ Treg cells from active BP patients were similar to those from healthy controls.^48^ The ratio of Foxp3^+^/CD4^+^ cells was higher in BP lesions than in control skin.^49^ The frequency of CD4^+^CD25^++^CD127^−^ Treg cells was significantly higher in the peripheral blood of BP patients than in that of healthy controls.^50^ In contrast, the frequency of circulating CD4^+^CD25^bright^Foxp3^+^ Treg cells was significantly lower in BP patients than in healthy controls.^51^ The frequencies of Foxp3^+^ cells and the numbers of IL‐10^+^ cells were significantly lower in skin lesions from BP patients than in those from patients with psoriasis or atopic dermatitis, suggesting a possible role of the reduction of Treg cells in the pathogenesis of BP.^52^ Thus, the results of Treg cells in the blood and skin lesions of BP patients were inconsistent. This inconsistency may be due to differences in methodologies for detecting human Tregs, the age of the studied subjects, or the stages and severities of the disease.

Miyara et al.^53^ demonstrated that human CD4^+^Foxp3^+^ T cells are divisible into three phenotypically and functionally distinct subpopulations: CD4^+^CD45RA^+^Foxp3^lo^ resting or naïve Treg cells (rTregs or nTregs), CD4^+^CD45RA^−^Foxp3^hi^ activated or effector Treg cells (aTregs or eTregs), and cytokine‐secreting CD4^+^CD45RA^−^Foxp3^lo^ nonsuppressive T cells (non‐Tregs). Of note, “non‐Tregs” express Foxp3 but have no immunosuppressive function, suggesting that we should carefully choose the method for detecting or isolating Treg cells when we analyze the frequency and function of Treg cells in humans. We recently investigated Treg cells and Treg subsets in the blood of BP patients using Miyara's method.^54^ We also investigated Treg cells in patients with DPP‐4i‐associated BP (DPP‐4i‐BP) and found that total Treg cells and all Treg subsets were increased in conventional BP (cBP) patients before treatment and were decreased by systemic corticosteroid treatment. Meanwhile, neither total Treg cells nor all Treg subsets were increased in DPP‐4i‐BP. Interestingly, CD45RA^+^Foxp3^hi^ effector Treg cells positively correlated with disease severity (BPDAI, blisters/erosions and erythema/urticaria) in cBP, whereas CD45RA^+^Foxp3^lo^ naïve Treg cells positively correlated with the disease severity (BPDAI, erythema/urticaria) in DPP‐4i‐BP. These results suggest that effector Treg cells with a suppressive function are expanded, possibly in response to the inflammation in active cBP, and that effector Treg cells suppress autoreactive T cells. We speculate that effector Treg cells cannot expand sufficiently in response to the autoreactive T cells in DPP‐4i‐BP, possibly because of the effect of DPP‐4i intake, resulting in a development of bullous lesions even in a mild inflammatory milieu.^54^

In summary, Treg cell dysfunction has some impact on the pathogenesis of BP, according to clinical findings from patients and to animal experiments. A further understanding of Treg properties by investigations with optimal experimental methods should lead to novel therapeutic strategies for BP.

## 5 BP TRIGGERING FACTORS

In considering the pathogenesis of BP, it is beneficial to understand the triggering factors. It is well known that BP is triggered by various factors such as trauma, burns, infections, radiotherapy, and ultraviolet exposure.^55^ Furthermore, the presence of neurological diseases such as dementia, stroke, and Parkinson's disease,^56^ hematological malignancies such as Hodgkin disease, nonfollicular lymphoma, mature T/NK‐cell lymphomas,^57^ and the use of DPP‐4i,^58^, ^59^ and immune checkpoint inhibitors (ICIs)^60^ were reported to increase the risk of BP onset. Here, we review those factors, focusing on physical triggers, neurological diseases, and ICIs.

### 5.1 Physical triggering factors

Mai et al.^55^ reported a case of BP triggered by thermal burns under medication with a DPP‐4i. In this report, a 60‐year‐old man with type II diabetes who had been treated with a DPP‐4i for 1 year experienced a thermal burn on the right forearm and then developed BP. This suggests that two risk factors for BP—the use of a DPP‐4i and a thermal burn—might have cooperatively or synergistically induced BP in that patient. In the study, physical triggering factors for BP were listed. In a total 147 cases, the top five factors were irradiation (*n* = 38), ultraviolet exposure (*n* = 37), surgical wound (*n* = 22), ostomy (*n* = 19), and burn (*n* = 14). Thus, various physical stresses can trigger BP. As the pathogenic mechanism behind the physical triggers, the Mai group hypothesized that the physical stresses cause tissue destruction that activates the inflammatory process, which may result in autoreactivity to basement membrane proteins, including COL17, or that basement membrane proteins may be altered as a result of physical factors resulting in immunogenicity with increased affinity to certain human leukocyte antigen (HLA) alleles.^55^

It is known that BP autoantibodies can be detected in a portion of BP patients before disease onset. BP autoantibodies that are detected in patients without typical tense blisters are defined as “preclinical BP autoantibodies”. These autoantibodies are detected even in a low percentage of normal healthy individuals, although it remains unclear how important preclinical BP autoantibodies are and whether the presence of anti‐COL17 autoantibodies are a predictive factor for BP development.^61^ Recently, Mai et al. examined sera from 1035 Japanese subjects by full‐length COL17 ELISA and found that 23 individuals (2.2%) possessed anti‐COL17 autoantibodies, but that none of the sera reacted with the NC16A domain of COL17 and that none of the 23 individuals developed BP.^62^ To identify the risk factors for anti‐COL17 autoantibody development, they examined the clinical records and found that anti‐COL17 autoantibodies are associated with a history of bone fracture and the administration of anti‐osteoporosis drugs. To determine the significance of the association, further study with a larger population is needed.

### 5.2 Neurological diseases

Previous epidemiological studies demonstrated that neurological diseases are a risk factor for BP.^56^, ^63^ Seppänen et al. reported COL17 to be expressed in many cells of the brain.^64^, ^65^ Anti‐COL17 autoantibodies were detected in 18% of patients who had Alzheimer's disease (AD) but did not have BP, whereas only 3% of controls were positive for these autoantibodies; notably, increased anti‐COL17 NC16A autoantibody values correlated with more severe dementia in AD, suggesting the association between BP and neurodegenerative diseases.^66^ Tuusa et al.^67^ demonstrated that 53.6% of sera from patients with multiple sclerosis (MS) had IgG autoantibodies to full‐length COL17 in immunoblotting. Most MS and AD sera reacted to various portions of COL17 (in denatured form) but not to the NC16A domain in immunoblotting, whereas more than half of BP sera reacted to the NC16A domain. Interestingly, in the ELISA using full‐length COL17 (in native form), 78.2% of the BP sera were positive, whereas only 11.4% of the MS samples, 6.3% of the AD samples, and 7.5% of the healthy control samples were positive. These findings suggest that autoantibodies in MS and AD sera target nonpathogenic epitopes of COL17 and preferentially react to the denatured form of COL17 but not to its native form.

### 5.3 Immune checkpoint inhibitors

Immunotherapies with immune checkpoint inhibitors (ICIs) that target programmed cell death 1 (PD‐1), programmed cell death ligand 1 (PD‐L1), and cytotoxic T‐lymphocyte‐associated protein 4 (CTLA‐4) have emerged as highly effective treatments for an increasing number of malignancies. Because the PD‐1 and CTLA‐4 pathways play important roles in regulating immune responses, their blockade leads to both generalized and organ‐specific inflammation called immune‐related adverse events (irAEs). The skin is a major target of irAE, presenting with nonspecific itchy maculopapular rashes, lichenoid reactions, psoriasis, acneiform rashes, vitiligo‐like lesions, autoimmune skin diseases (e.g., dermatomyositis, alopecia areata), sarcoidosis, or nail and oral mucosal changes.^68^ BP is a well‐known skin irAEs whose frequency is reported to range from 0.3% to 0.8% among patients under ICI treatment,^60^, ^69^, ^70^ which far exceeds the incidence of BP in the general population (0.0012%–0.0076%).^71^, ^72^, ^73^, ^74^, ^75^, ^76^ Siegel et al.^70^ reported seven cases of BP associated with ICI therapy (ICI‐BP) as well as a case of bullous lichenoid dermatitis and a case of linear IgA bullous dermatosis, and they showed that the time from therapy initiation to rash was variable, with a mean latency of 6.25 months and a range of 2 weeks to 20 months. All nine patients had either an initial positive tumor response or stable disease, although six eventually developed disease progression. In all cases, ICIs were discontinued or interrupted. Eight of the nine patients required systemic steroids for the treatment of rash. Apalla et al. summarized 13 cases of ICI‐BP and showed that ICI was unaltered in seven, temporarily interrupted in two, and permanently discontinued in four, and seven patients who continued ICI remained free of BP with an average prednisolone dose of 2.5–5 mg/day.^77^ The authors proposed that grade‐1 or grade‐2 eruptions can be managed with low doses (0.3–0.5 mg/kg/day) of prednisolone and potent topical steroids, without impeding the administration of ICI. In more severe cases, treatment starts with a low dose of prednisolone without altering the ICI, and if there is no response, BP can be controlled by increasing the prednisolone to 0.7 mg/kg/day while withholding one or tw doses of the ICI. Juzot et al.^78^ reported 85 ICI‐BP cases. In those cases, the first‐line therapy for BP was topical steroids (the whole‐body application of topical clobetasol) in 84% (*n* = 71), systemic corticosteroids in 5%, topical and systemic corticosteroids in 8%, and topical steroids and doxycycline in 2%. The ICI was permanently discontinued for 40 patients. Seventy‐one patients were treated with topical steroids as monotherapy, and 31 patients discontinued the ICI while 39 continued it. Of note, among those who discontinued the ICI, 17 (55%) had their BP controlled, whereas among those who continued the ICI, 29 (74%) had their BP controlled.^78^ These results show that high‐potency topical steroids may be sufficient to control BP even with continuing ICIs in some cases.

A recent systematic review of 70 studies on 127 ICI‐BP cases^79^ showed that ICI‐BP often occurred during immunotherapy but was also found to develop up to several months after treatment cessation. About half of the patients experienced prodromal symptoms before the development of BP, with the most common symptom being pruritus or largely nonspecific erythematous skin eruptions. Anti‐COL17 autoantibodies were detected in 46 of 57 patients (80.7%), whereas anti‐BP230 autoantibodies were found in only nine of 31 patients (29.0%). Among patients with reported data (*n* = 120), the ICI treatment was discontinued immediately after BP development in 63 patients (52.5%) and after a failed attempt at ICI continuation in seven patients (5.8%). In patients with treatment data (*n* = 126), oral or intravenous corticosteroids (*n* = 107, 84.9%) with a wide range of doses were the most commonly used treatment modalities, followed by topical steroids (*n* = 68, 54.0%). Furthermore, 42.1% of patients (*n* = 53) required treatment with systemic and topical corticosteroids, whereas 12.7% (*n* = 16) were treated with topical steroids only. Other nonsteroidal treatments, such as antibiotics, rituximab, and omalizumab, were additionally used in some cases. It was found that 81.4% of patients (96 of 118) experienced partial to complete improvement of BP with treatment.

Thus, the necessity of ICI discontinuation and the necessity of systemic corticosteroids for BP remain controversial. Considering the patient's condition, lower doses of systemic corticosteroids and the continuation of ICIs are desirable for most cases. To optimize the treatment algorithm for ICI‐BP, the effects of topical steroids, steroid‐sparing agents, and immunomodulatory therapies should be further investigated.

## 6 CONCLUSION

There are two major focuses of BP research: (1) the mechanism behind the breakdown of immune tolerance to BP antigens (COL17 and BP230) and (2) the molecular and immunological pathways of blister formation and inflammation triggered by BP autoantibodies. The latter has been thoroughly investigated based on animal models and patient samples, and the complement‐dependent and ‐independent pathways have been well elucidated.^80^ As shown in this review, an improved understanding of the epitope‐spreading phenomenon and the pathogenicity of anti‐BP230 autoantibodies may elucidate the mechanism behind disease progression and the chronic disease course of BP. In addition, type 2 immune reactions, eosinophils, mast cells, granzymes, IgE autoantibodies, and Fc receptors have been attracting researchers' interest. Meanwhile, the mechanism behind the breakdown of immune tolerance to BP antigens remains largely unclear. Not only might Treg cells give us clues for understanding the underlying mechanisms, but so might research on central tolerance, triggering factors, and immunosenescence.

**FIGURE 4:**
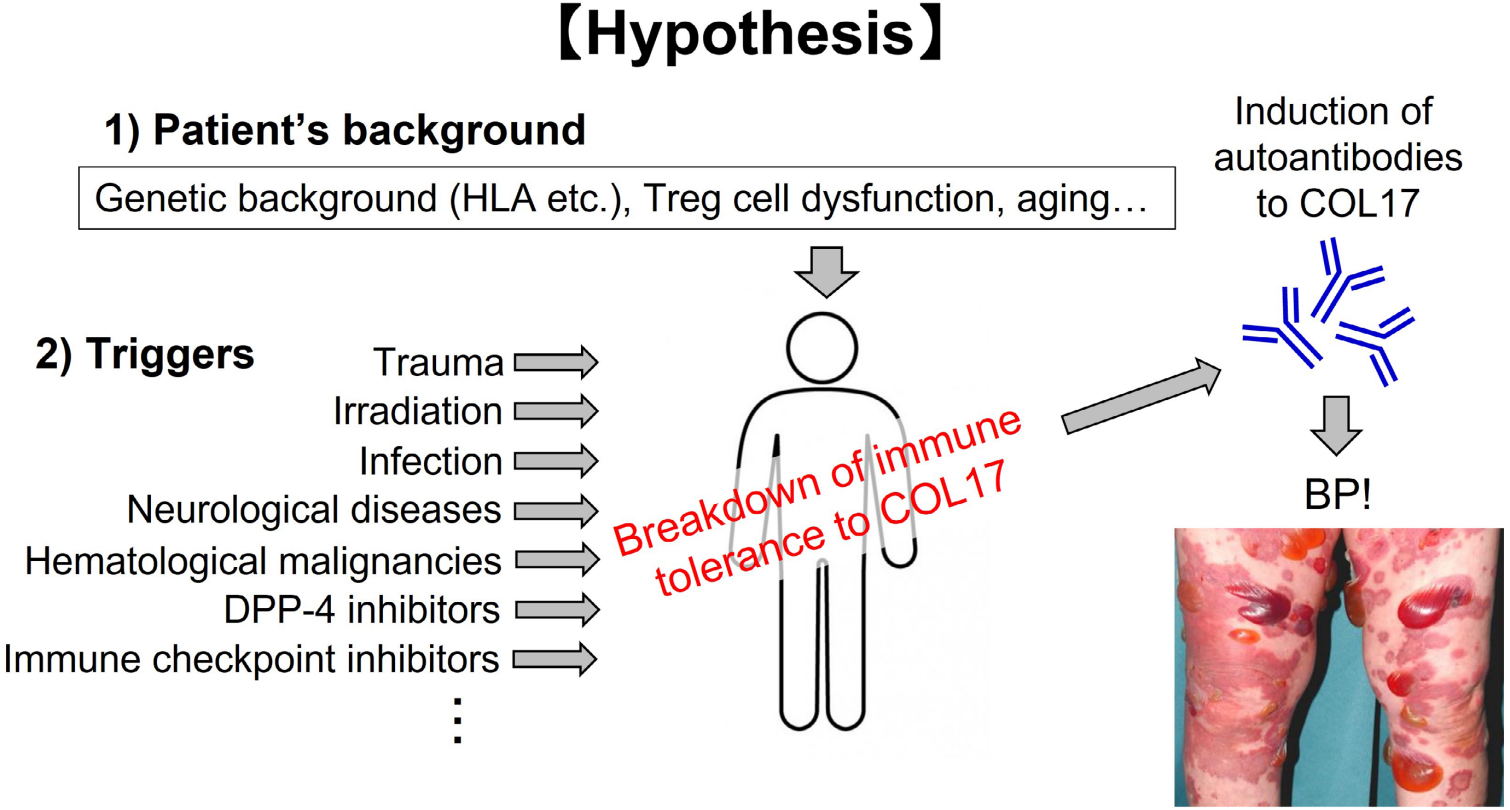
Our hypothesis on the pathogenesis of bullous pemphigoid (BP). We hypothesize that the patient's background, such as genetic background, Treg cell dysfunction and aging, as well as triggers such as trauma, irradiation, infection, neurological diseases, hematological malignancies, DPP‐4 inhibitors, immune checkpoint inhibitors, and the like, cooperatively or synergistically induce the breakdown of immune tolerance to COL17, resulting in the production of autoantibodies and the development of BP.

Figure 4 shows our hypothesis of the pathogenesis of BP. The patient's background—e.g., genetic background (such as HLA‐DQB1*03:01 in Asian DPP‐4i‐BP patients^81^, ^82^), Treg cell dysfunction, and aging—as well as triggering factors—e.g., trauma, irradiation, infection, neurological diseases, hematological malignancies, drugs (such as DPP‐4i and ICIs)—cooperatively or synergistically induce the breakdown of immune tolerance to COL17 and result in the production of autoantibodies and the onset of BP. A further understanding of the underlying mechanisms will be helpful in developing less harmful, more disease‐specific therapeutic approaches for BP.

## CONFLICT OF INTEREST

The authors declare that we have no conflicts of interest.

## References

[1] Kridin K , Ludwig RJ . The growing incidence of bullous pemphigoid: overview and potential explanations. Front Med. 2018;5:220. 10.3389/fmed.2018.00220 PMC6109638 30177969

[2] Ujiie H , Iwata H , Yamagami J , Nakama T , Aoyama Y , Ikeda S , et al. Japanese guidelines for the management of pemphigoid (including epidermolysis bullosa acquisita). J Dermatol. 2019;46:1102–35. 31646663 10.1111/1346-8138.15111

[3] Schmidt E , Zillikens D . Pemphigoid diseases. Lancet. 2013;381:320–32. 23237497 10.1016/S0140-6736(12)61140-4

[4] Diaz LA , Ratrie H , Saunders WS , Futamura S , Squiquera HL , Anhalt GJ , et al. Isolation of a human epidermal cDNA corresponding to the 180‐kD autoantigen recognized by bullous pemphigoid and herpes gestationis sera. Immunolocalization of this protein to the hemidesmosome. J Clin Invest. 1990;86:1088–94. 1698819 10.1172/JCI114812 PMC296836

[5] Giudice GJ , Emery DJ , Diaz LA . Cloning and primary structural analysis of the bullous pemphigoid autoantigen BP180. J Invest Dermatol. 1992;99:243–50. 1324962 10.1111/1523-1747.ep12616580

[6] Ishiko A , Shimizu H , Kikuchi A , Ebihara T , Hashimoto T , Nishikawa T . Human autoantibodies against the 230‐kD bullous pemphigoid antigen (BPAG1) bind only to the intracellular domain of the hemidesmosome, whereas those against the 180‐kD bullous pemphigoid antigen (BPAG2) bind along the plasma membrane of the hemidesmosome. J Clin Invest. 1993;91:1608–15. 7682575 10.1172/JCI116368 PMC288138

[7] Matsumura K , Amagai M , Nishikawa T , Hashimoto T . The majority of bullous pemphigoid and herpes gestationis serum samples react with the NC16a domain of the 180‐kDa bullous pemphigoid antigen. Arch Dermatol Res. 1996;288:507–9. 8874743 10.1007/BF02505245

[8] Zillikens D , Rose PA , Balding SD , Liu Z , Olague‐Marchan M , Diaz LA , et al. Tight clustering of extracellular BP180 epitopes recognized by bullous pemphigoid autoantibodies. J Invest Dermatol. 1997;109:573–9. 9326393 10.1111/1523-1747.ep12337492

[9] Zillikens D , Mascaro JM , Rose PA , Liu Z , Ewing SM , Caux F , et al. A highly sensitive enzyme‐linked immunosorbent assay for the detection of circulating anti‐BP180 autoantibodies in patients with bullous pemphigoid. J Invest Dermatol. 1997;109:679–83. 9347799 10.1111/1523-1747.ep12338088

[10] Kobayashi M , Amagai M , Kuroda‐Kinoshita K , Hashimoto T , Shirakata Y , Hashimoto K , et al. BP180 ELISA using bacterial recombinant NC16a protein as a diagnostic and monitoring tool for bullous pemphigoid. J Dermatol Sci. 2002;30:224–32. 12443845 10.1016/s0923-1811(02)00109-3

[11] Tsuji‐Abe Y , Akiyama M , Yamanaka Y , Kikuchi T , Sato‐Matsumura KC , Shimizu H . Correlation of clinical severity and ELISA indices for the NC16A domain of BP180 measured using BP180 ELISA kit in bullous pemphigoid. J Dermatol Sci. 2005;37:145–9. 15734283 10.1016/j.jdermsci.2004.10.007

[12] Döpp R , Schmidt E , Chimanovitch I , Leverkus M , Bröcker EB , Zillikens D . IgG4 and IgE are the major immunoglobulins targeting the NC16A domain of BP180 in bullous pemphigoid: serum levels of these immunoglobulins reflect disease activity. J Am Acad Dermatol. 2000;42:577–83. 10727301

[13] Liu Z , Diaz LA , Troy JL , Taylor AF , Emery DJ , Fairley JA , et al. A passive transfer model of the organ‐specific autoimmune disease, bullous pemphigoid, using antibodies generated against the hemidesmosomal antigen, BP180. J Clin Invest. 1993;92:2480–8. 7693763 10.1172/JCI116856 PMC288433

[14] Nishie W , Sawamura D , Goto M , Ito K , Shibaki A , McMillan JR , et al. Humanization of autoantigen. Nat Med. 2007;13:378–83. 17322897 10.1038/nm1496

[15] Izumi K , Nishie W , Mai Y , Wada M , Natsuga K , Ujiie H , et al. Autoantibody profile differentiates between inflammatory and noninflammatory bullous pemphigoid. J Invest Dermatol. 2016;136:2201–10. 27424319 10.1016/j.jid.2016.06.622

[16] Blöcker IM , Dähnrich C , Probst C , Komorowski L , Saschenbrecker S , Schlumberger W , et al. Epitope mapping of BP230 leading to a novel enzyme‐linked immunosorbent assay for autoantibodies in bullous pemphigoid. Br J Dermatol. 2012;166:964–70. 22242606 10.1111/j.1365-2133.2012.10820.x

[17] Skaria M , Jaunin F , Hunziker T , Riou S , Schumann H , Bruckner‐Tuderman L , et al. IgG autoantibodies from bullous pemphigoid patients recognize multiple antigenic reactive sites located predominantly within the B and C subdomains of the COOH‐terminus of BP230. J Invest Dermatol. 2000;114:998–1004. 10771483 10.1046/j.1523-1747.2000.00893.x

[18] Thoma‐Uszynski S , Uter W , Schwietzke S , Hofmann SC , Hunziker T , Bernard P , et al. BP230‐ and BP180‐specific auto‐antibodies in bullous pemphigoid. J Invest Dermatol. 2004;122:1413–22. 15175032 10.1111/j.0022-202X.2004.22603.x

[19] Hall RP , Murray JC , MM MC , Rico MJ , Streilein RD . Rabbits immunized with a peptide encoded for by the 230‐kD bullous pemphigoid antigen cDNA develop an enhanced inflammatory response to UVB irradiation: a potential animal model for bullous pemphigoid. J Invest Dermatol. 1993;101:9–14. 8331301 10.1111/1523-1747.ep12358276

[20] Kiss M , Husz S , Jánossy T , Marczinovits I , Molnár J , Korom I , et al. Experimental bullous pemphigoid generated in mice with an antigenic epitope of the human hemidesmosomal protein BP230. J Autoimmun. 2005;24:1–10. 15725571 10.1016/j.jaut.2004.09.007

[21] Chan LS , Vanderlugt CJ , Hashimoto T , Nishikawa T , Zone JJ , Black MM , et al. Epitope spreading: lessons from autoimmune skin diseases. J Invest Dermatol. 1998;110:103–9. 9457902 10.1046/j.1523-1747.1998.00107.x

[22] Didona D , Di Zenzo G . Humoral epitope spreading in autoimmune bullous diseases. Front Immunol. 2018;9:779. 29719538 10.3389/fimmu.2018.00779 PMC5913575

[23] Tuohy VK , Yu M , Yin L , Kawczak JA , Philip Kinkel R . Spontaneous regression of primary autoreactivity during chronic progression of experimental autoimmune encephalomyelitis and multiple sclerosis. J Exp Med. 1999;189:1033–42. 10190894 10.1084/jem.189.7.1033 PMC2193005

[24] Huijbers MG , Vink A‐FD , Niks EH , Westhuis RH , van Zwet EW , de Meel RH , et al. Longitudinal epitope mapping in MuSK myasthenia gravis: implications for disease severity. J Neuroimmunol. 2016;291:82–8. 26857500 10.1016/j.jneuroim.2015.12.016

[25] Di Zenzo G , Calabresi V , Olasz EB , Zambruno G , Yancey KB . Sequential intramolecular epitope spreading of humoral responses to human BPAG2 in a transgenic model. J Invest Dermatol. 2010;130:1040–7. 19812601 10.1038/jid.2009.309

[26] Di Zenzo G , Thoma‐Uszynski S , Calabresi V , Fontao L , Hofmann SC , Lacour J‐P , et al. Demonstration of epitope‐spreading phenomena in bullous pemphigoid: results of a prospective multicenter study. J Invest Dermatol. 2011;131:2271–80. 21697892 10.1038/jid.2011.180

[27] Ujiie H , Shibaki A , Nishie W , Sawamura D , Wang G , Tateishi Y , et al. A novel active mouse model for bullous pemphigoid targeting humanized pathogenic antigen. J Immunol. 2010;184:2166–74. 20089696 10.4049/jimmunol.0903101

[28] Ujiie H , Yoshimoto N , Natsuga K , Muramatsu K , Iwata H , Nishie W , et al. Immune reaction to type XVII collagen induces intramolecular and intermolecular epitope spreading in experimental bullous pemphigoid models. Front Immunol. 2019;10:1410. 31275329 10.3389/fimmu.2019.01410 PMC6593113

[29] Seo T , Ujiie H , Ujiie I , Iwata H , Shimizu H . Epitope spreading possibly from BP230 to the NC16A domain of BP180 preceding disease progression in bullous pemphigoid. J Dermatol. 2020;47:e255–7. 32343019 10.1111/1346-8138.15362

[30] Haeberle S , Wei X , Bieber K , Goletz S , Ludwig RJ , Schmidt E , et al. Regulatory T‐cell deficiency leads to pathogenic bullous pemphigoid antigen 230 autoantibody and autoimmune bullous disease. J Allergy Clin Immunol. 2018;142:1831–42.e7. 29704595 10.1016/j.jaci.2018.04.006

[31] Makita E , Matsuzaki Y , Fukui T , Matsui A , Minakawa S , Nakano H , et al. Autoantibodies to BPAG1e trigger experimental bullous pemphigoid in mice. J Invest Dermatol. 2021;141:1167–76.e3. 33069726 10.1016/j.jid.2020.08.031

[32] Hayakawa T , Teye K , Hachiya T , Uehara R , Hashiguchi M , Kawakami T , et al. Clinical and immunological profiles of anti‐BP230‐type bullous pemphigoid: restriction of epitopes to the C‐terminal domain of BP230, shown by novel ELISAs of BP230‐domain specific recombinant proteins. Eur J Dermatol. 2016;26:155–63. 27087683 10.1684/ejd.2015.2719

[33] Zheng M , Ujiie H , Iwata H , Muramatsu K , Yoshimoto N , Ito T , et al. Characteristics of IgG subclasses and complement deposition in BP230‐type bullous pemphigoid. J Eur Acad Dermatol Venereol. 2019;33:595–600. 30394605 10.1111/jdv.15325

[34] Yoshimoto N , Ujiie I , Inamura E , Natsuga K , Nishie W , Shimizu H , et al. A case of mucous membrane pemphigoid with anti‐BP230 autoantibodies alone. Int J Dermatol. 2021;60:e92–4. 32970829 10.1111/ijd.15195

[35] Yoshimoto N , Takashima S , Kawamura T , Inamura E , Sugai T , Ujiie I , et al. A case of non‐bullous pemphigoid induced by IgG4 autoantibodies targeting BP230. J Eur Acad Dermatol Venereol. 2021;35:e282–5. 33219610 10.1111/jdv.17044

[36] Akkaya B , Shevach EM . Regulatory T cells: master thieves of the immune system. Cell Immunol. 2020;355:104160. 32711171 10.1016/j.cellimm.2020.104160 PMC9761694

[37] Bennett CL , Christie J , Ramsdell F , Brunkow ME , Ferguson PJ , Whitesell L , et al. The immune dysregulation, polyendocrinopathy, enteropathy, X‐linked syndrome (IPEX) is caused by mutations of FOXP3. Nat Genet. 2001;27:20–1. 11137993 10.1038/83713

[38] Iellem A , Mariani M , Lang R , Recalde H , Panina‐Bordignon P , Sinigaglia F , et al. Unique chemotactic response profile and specific expression of chemokine receptors CCR4 and CCR8 by CD4(+)CD25(+) regulatory T cells. J Exp Med. 2001;194:847–53. 11560999 10.1084/jem.194.6.847 PMC2195967

[39] Wildin RS , Ramsdell F , Peake J , Faravelli F , Casanova JL , Buist N , et al. X‐linked neonatal diabetes mellitus, enteropathy and endocrinopathy syndrome is the human equivalent of mouse scurfy. Nat Genet. 2001;27:18–20. 11137992 10.1038/83707

[40] Rosenblum MD , Gratz IK , Paw JS , Lee K , Marshak‐Rothstein A , Abbas AK . Response to self antigen imprints regulatory memory in tissues. Nature. 2011;480:538–42. 22121024 10.1038/nature10664 PMC3263357

[41] Nosbaum A , Prevel N , Truong H‐A , Mehta P , Ettinger M , Scharschmidt TC , et al. Cutting edge: regulatory T cells facilitate cutaneous wound healing. J Immunol. 2016;196:2010–4. 26826250 10.4049/jimmunol.1502139 PMC4761457

[42] Scharschmidt TC , Vasquez KS , Truong HA , Gearty SV , Pauli ML , Nosbaum A , et al. A wave of regulatory T cells into neonatal skin mediates tolerance to commensal microbes. Immunity. 2015;43:1011–21. 26588783 10.1016/j.immuni.2015.10.016 PMC4654993

[43] Ali N , Zirak B , Rodriguez RS , Pauli ML , Truong HA , Lai K , et al. Regulatory T cells in skin facilitate epithelial stem cell differentiation. Cell. 2017;169:1119–29.e11. 28552347 10.1016/j.cell.2017.05.002 PMC5504703

[44] Mathur AN , Zirak B , Boothby IC , Tan M , Cohen JN , Mauro TM , et al. Treg‐cell control of a CXCL5‐IL‐17 inflammatory Axis promotes hair‐follicle‐stem‐cell differentiation during skin‐barrier repair. Immunity. 2019;50:655–67.e4. 30893588 10.1016/j.immuni.2019.02.013 PMC6507428

[45] Ujiie H . Regulatory T cells in autoimmune skin diseases. Exp Dermatol. 2019;28:642–6. 29575350 10.1111/exd.13535

[46] Muramatsu K , Ujiie H , Kobayashi I , Nishie W , Izumi K , Ito T , et al. Regulatory T‐cell dysfunction induces autoantibodies to bullous pemphigoid antigens in mice and human subjects. J Allergy Clin Immunol. 2018;142:1818–30.e6. 29704593 10.1016/j.jaci.2018.03.014

[47] Bieber K , Sun S , Witte M , Kasprick A , Beltsiou F , Behnen M , et al. Regulatory T cells suppress inflammation and blistering in pemphigoid diseases. Front Immunol. 2017;8:1628. 29225603 10.3389/fimmu.2017.01628 PMC5705561

[48] Rensing‐Ehl A , Gaus B , Bruckner‐Tuderman L , Martin SF . Frequency, function and CLA expression of CD4+CD25+FOXP3+ regulatory T cells in bullous pemphigoid. Exp Dermatol. 2007;16:13–21. 17181632 10.1111/j.1600-0625.2006.00522.x

[49] Arakawa M , Dainichi T , Ishii N , Hamada T , Karashima T , Nakama T , et al. Lesional Th17 cells and regulatory T cells in bullous pemphigoid. Exp Dermatol. 2011;20:1022–4. 22017210 10.1111/j.1600-0625.2011.01378.x

[50] Gambichler T , Tsitlakidon A , Skrygan M , Höxtermann S , Susok L , Hessam S . T regulatory cells and other lymphocyte subsets in patients with bullous pemphigoid. Clin Exp Dermatol. 2017;42:632–7. 28590036 10.1111/ced.13135

[51] Quaglino P , Antiga E , Comessatti A , Caproni M , Nardò T , Ponti R , et al. Circulating CD4+ CD25brightFOXP3+ regulatory T‐cells are significantly reduced in bullous pemphigoid patients. Arch Dermatol Res. 2012;304:639–45. 22310732 10.1007/s00403-012-1213-9

[52] Antiga E , Quaglino P , Volpi W , Pierini I , del Bianco E , Bianchi B , et al. Regulatory T cells in skin lesions and blood of patients with bullous pemphigoid. J Eur Acad Dermatol Venereol. 2014;28:222–30. 23331964 10.1111/jdv.12091

[53] Miyara M , Yoshioka Y , Kitoh A , Shima T , Wing K , Niwa A , et al. Functional delineation and differentiation dynamics of human CD4+ T cells expressing the FoxP3 transcription factor. Immunity. 2009;30:899–911. 19464196 10.1016/j.immuni.2009.03.019

[54] Muramatsu K , Zheng M , Yoshimoto N , Ito T , Ujiie I , Iwata H , et al. Regulatory T cell subsets in bullous pemphigoid and dipeptidyl peptidase‐4 inhibitor‐associated bullous pemphigoid. J Dermatol Sci. 2020;100:23–30. 32843228 10.1016/j.jdermsci.2020.08.004

[55] Mai Y , Nishie W , Sato K , Hotta M , Izumi K , Ito K , et al. Bullous pemphigoid triggered by thermal burn under medication with a dipeptidyl peptidase‐IV inhibitor: a case report and review of the literature. Front Immunol. 2018;9:542. 29706950 10.3389/fimmu.2018.00542 PMC5906537

[56] Langan SM , Groves RW , West J . The relationship between neurological disease and bullous pemphigoid: a population‐based case‐control study. J Invest Dermatol. 2011;131:631–6. 21085189 10.1038/jid.2010.357

[57] Schulze F , Neumann K , Recke A , Zillikens D , Linder R , Schmidt E . Malignancies in pemphigus and pemphigoid diseases. J Invest Dermatol. 2015;135:1445–7. 25560279 10.1038/jid.2014.547

[58] Béné J , Moulis G , Bennani I , Auffret M , Coupe P , Babai S , et al. Bullous pemphigoid and dipeptidyl peptidase IV inhibitors: a case‐noncase study in the French Pharmacovigilance database. Br J Dermatol. 2016;175:296–301. 27031194 10.1111/bjd.14601

[59] Arai M , Shirakawa J , Konishi H , Sagawa N , Terauchi Y . Bullous pemphigoid and dipeptidyl peptidase 4 inhibitors: a disproportionality analysis based on the Japanese adverse drug event report database. Diabetes Care. 2018;41:e130–2. 30002201 10.2337/dc18-0210

[60] Kawsar A , Edwards C , Patel P , Heywood RM , Gupta A , Mann J , et al. Checkpoint inhibitor associated bullous cutaneous immune related adverse events: a multi‐centre observational study. Br J Dermatol. 2022; online ahead of print. 10.1111/bjd.21836 35976170

[61] Mai Y , Izumi K , Mai S , Ujiie H . The significance of preclinical anti‐BP180 autoantibodies. Front Immunol. 2022;13:963401. 36003369 10.3389/fimmu.2022.963401 PMC9393388

[62] Mai Y , Izumi K , Sawada K , Akasaka E , Mai S , Sawamura D , et al. A 1,035‐subject study suggesting a history of bone fracture as a possible factor associated with the development of anti‐BP180 autoantibodies. J Invest Dermatol. 2022;142:984–7.e3. 34883045 10.1016/j.jid.2021.11.028

[63] Taghipour K , Chi C‐C , Bhogal B , Groves RW , Venning V , Wojnarowska F . Immunopathological characteristics of patients with bullous pemphigoid and neurological disease. J Eur Acad Dermatol Venereol. 2014;28:569–73. 23530989 10.1111/jdv.12136

[64] Seppänen A , Autio‐Harmainen H , Alafuzoff I , Särkioja T , Veijola J , Hurskainen T , et al. Collagen XVII is expressed in human CNS neurons. Matrix Biol. 2006;25:185–8. 16387484 10.1016/j.matbio.2005.11.004

[65] Seppänen A , Suuronen T , Hofmann SC , Majamaa K , Alafuzoff I . Distribution of collagen XVII in the human brain. Brain Res. 2007;1158:50–6. 17555727 10.1016/j.brainres.2007.04.073

[66] Kokkonen N , Herukka SK , Huilaja L , Kokki M , Koivisto AM , Hartikainen P , et al. Increased levels of the bullous pemphigoid BP180 autoantibody are associated with more severe dementia in Alzheimer's disease. J Invest Dermatol. 2017;137:71–6. 27650606 10.1016/j.jid.2016.09.010

[67] Tuusa J , Lindgren O , Tertsunen HM , Nishie W , Kokkonen N , Huilaja L , et al. BP180 autoantibodies target different epitopes in multiple sclerosis or Alzheimer's disease than in bullous pemphigoid. J Invest Dermatol. 2019;139:293–9. 30315782 10.1016/j.jid.2018.09.010

[68] Sibaud V . Dermatologic reactions to immune checkpoint inhibitors: skin toxicities and immunotherapy. Am J Clin Dermatol. 2018;19:345–61. 29256113 10.1007/s40257-017-0336-3

[69] Nelson CA , Singer S , Chen T , Puleo AE , Lian CG , Wei EX , et al. Bullous pemphigoid after anti‐PD‐1 therapy: a retrospective case‐control study evaluating impact on tumor response and survival outcomes. J Am Acad Dermatol. 2020; online ahead of print.

[70] Siegel J , Totonchy M , Damsky W , Berk‐Krauss J , Castiglione F Jr , Sznol M , et al. Bullous disorders associated with anti–PD‐1 and anti–PD‐L1 therapy: a retrospective analysis evaluating the clinical and histopathologic features, frequency, and impact on cancer therapy. J Am Acad Dermatol. 2018;79:1081–8. 30025829 10.1016/j.jaad.2018.07.008

[71] Marazza G , Pham HC , Schärer L , Pedrazzetti PP , Hunziker T , Trüeb RM , et al. Incidence of bullous pemphigoid and pemphigus in Switzerland: a 2‐year prospective study. Br J Dermatol. 2009;161:861–8. 19566661 10.1111/j.1365-2133.2009.09300.x

[72] Langan SM , Smeeth L , Hubbard R , Fleming KM , Smith CJP , West J . Bullous pemphigoid and pemphigus vulgaris – incidence and mortality in the UK: population based cohort study. BMJ. 2008;337:a180. 18614511 10.1136/bmj.a180 PMC2483869

[73] Bertram F , Bröcker E‐B , Zillikens D , Schmidt E . Prospective analysis of the incidence of autoimmune bullous disorders in lower Franconia, Germany. J Dtsch Dermatol Ges. 2009;7:434–40. 19170813 10.1111/j.1610-0387.2008.06976.x

[74] Gudi VS , White MI , Cruickshank N , Herriot R , Edwards SL , Nimmo F , et al. Annual incidence and mortality of bullous pemphigoid in the Grampian region of north‐east Scotland. Br J Dermatol. 2005;153:424–7. 16086760 10.1111/j.1365-2133.2005.06662.x

[75] Joly P , Baricault S , Sparsa A , Bernard P , Bédane C , Duvert‐Lehembre S , et al. Incidence and mortality of bullous pemphigoid in France. J Invest Dermatol. 2012;132:1998–2004. 22418872 10.1038/jid.2012.35

[76] Persson MSM , Harman KE , Vinogradova Y , Langan SM , Hippisley‐Cox J , Thomas KS , et al. Incidence, prevalence and mortality of bullous pemphigoid in England 1998–2017: a population‐based cohort study. Br J Dermatol. 2021;184:68–77. 32147814 10.1111/bjd.19022

[77] Apalla Z , Lallas A , Delli F , Lazaridou E , Papalampou S , Apostolidou S , et al. Management of immune checkpoint inhibitor–induced bullous pemphigoid. J Am Acad Dermatol. 2021;84:540–3. 32428613 10.1016/j.jaad.2020.05.045

[78] Juzot C , Sibaud V , Amatore F , Mansard S , Seta V , Jeudy G , et al. Clinical, biological and histological characteristics of bullous pemphigoid associated with anti‐PD‐1/PD‐L1 therapy: a national retrospective study. J Eur Acad Dermatology Venereol. 2021;35:e511–4. 10.1111/jdv.17253 33783881

[79] Asdourian MS , Shah N , Jacoby TV , Reynolds KL , Chen ST . Association of bullous pemphigoid with immune checkpoint inhibitor therapy in patients with cancer: a systematic review. JAMA Dermatol. 2022;158:933–41. 35612829 10.1001/jamadermatol.2022.1624

[80] Papara C , Karsten CM , Ujiie H , Schmidt E , Schmidt‐Jiménez LF , Baican A , et al. The relevance of complement in pemphigoid diseases: a critical appraisal. Front Immunol. 2022;13:973702. 36059476 10.3389/fimmu.2022.973702 PMC9434693

[81] Ujiie H , Muramatsu K , Mushiroda T , Ozeki T , Miyoshi H , Iwata H , et al. HLA‐DQB1*03:01 as a biomarker for genetic susceptibility to bullous pemphigoid induced by DPP‐4 inhibitors. J Invest Dermatol. 2018;138:1201–4. 29203362 10.1016/j.jid.2017.11.023

[82] Chanprapaph K , Pratumchart N , Limtong P , Rutnin S , Sukasem C , Kungvalpivat P , et al. Dipeptidyl peptidase‐4 inhibitor‐related bullous pemphigoid: a comparative study of 100 patients with bullous pemphigoid and diabetes mellitus. J Dermatol. 2021;48:486–96. 33543537 10.1111/1346-8138.15778

